# Modification of pomegranate waste with iron ions a green composite for removal of Pb from aqueous solution: equilibrium, thermodynamic and kinetic studies

**DOI:** 10.1186/s13568-017-0520-0

**Published:** 2017-12-22

**Authors:** Mohammad Hossein Salmani, Mohammad Abedi, Sayed Ahmad Mozaffari, Hossien Ali Sadeghian

**Affiliations:** 1 Department of Environmental Health, School of Public Health, Shahid Sadoughi University of Medical Science, Yazd, Islamic Republic of Iran; 2 Department of Chemical Technologies, Iranian Research Organization for Science and Technology (IROST), Tehran, Islamic Republic of Iran; 3 Department of Environmental Health Education and Health Promotion, School of Public Health, Shahid Sadoughi University of Medical Sciences, Yazd, Islamic Republic of Iran

**Keywords:** Iron modified carbon, Pomegranate peels, Composite, Thermodynamic, Kinetic

## Abstract

Pomegranate waste modified with Fe^2+^ and Fe^3+^ ions followed with carbonization were used as an adsorbent to remove the Pb^2+^ ions from aqueous solution. To optimum the highest adsorption efficiency, adsorption experiments were conducted on iron modified carbons by batch technique. The characteristic of composite was studied by scanning electron microscope (SEM) and Fourier transform infrared spectrometer (FT-IR). The best pH for control of chemical adsorption was selected within pH of 6.0–6.5. It was observed that the contact time of 90 min, initial concentration 50.0 ppm, and adsorbent dose, 1.0 g/100 ml solution was found to be optimum conditions. On this condition, the maximum adsorption capacity was obtained 27.5 and 22.5 mg/g for Fe^2+^ and Fe^3+^ impregnated pomegranate peel carbons (PPC), respectively. The value of C_id_, 1.584 for Fe^2+^-PPC and 0.552 for Fe^3+^-PPC, indicates that the effect of the boundary layer is more important in adsorption of Pb^2+^ by Fe^2+^-PPC and the pore diffusion is the rate limiting mechanism after 30 min. Thermodynamic parameters of Gibbs free energy, enthalpy, and entropy of Pb^2+^ adsorption on iron-modified carbons suggest that the adsorption process is favorable and spontaneous under the optimum condition.

## Introduction

Lead is known as the heavy metals, which is strongly toxic to humans, animals, and plants. Inter of lead to the human body even at low concentrations causes serious problems to the nervous and reproductive system, kidney, liver, brain and bony tissues (Grant 2008; Renner 2010; Andrade et al. 2015). Environmental Protection Agency (EPA) approved the maximum threshold limit of 0.015 ppm for lead in drinking water. The removal of lead from wastewaters, before discharging to aquatic environment, in high concentration is accomplished by common processes, including precipitation with hydroxide ion or lime and in low concentration by advanced processes such as ion exchange, coagulation, electrochemical process, reverse osmosis, and ion flotation (Ehrampoush et al. 2015). These methods are costly and produce the secondary wastes. One of the high-performance and low-cost methods for removing of ionic pollutants from aqueous solutions is adsorption. However, the cost of used adsorbents and their separation from suspension after adsorption are the important restricting factors in perspective on the applicability of adsorption process. Recently, considerable notation has been given to the development of substitutes from readily available and cheaper materials such as agricultural wastes for preparation of activated carbon to remove pollutants to approach the standard levels (Babel and Kurniawan 2003). Until now, using of low-cost agricultural sorbents, including *Ponkan peel* (Pavan et al. 2008), waste *tea leaves* (Cheraghi et al. 2015), *potato peel* (Taha et al. 2011), modified *onion skins* (Saka et al. 2011), the activate carbon of *peanut shell* (Wilson et al. 2006), *pistachio shell*, and *apricot stone* (Kazemipour et al. 2008), have been examined for removing of Pb^2+^ ions from aqueous solutions.

Pomegranate wastes as a by-product of juice, jams, syrup, and sauce manufacturing industries, which produced in large amounts in Iran. The peels include about 45–50% of the total weight of fruit. The fruit peels have a strong affinity and high selection towards heavy metals because of the various functional groups on the surface of it. Hence, the idea to change the by-product of pomegranate fruit to iron impregnation carbon for cleaning the environment and removing of heavy metal is valuable. Abedi et al. impregnated of Fe^2+^ and Fe^3+^ ions to the PPC were studied to remove Cd(II) ions (Abedi et al. 2016).

Considering the above descriptions, we used as an adsorbent the iron modification of PPCs for removing of Pb^2+^ from aqueous solution by one factor at a time methodology. The main objective of this work is to obtain the adsorption kinetic and thermodynamic parameters for the adsorption reaction of Pb^2+^ ions with prepared composite.

The adsorption process depends on many factors including pH, temperature, adsorbent mass, concentration of pollutant, adsorbent type, contact time and agitation time. In the present study, the effect of initial lead concentration, adsorbent dose, and temperature was screened for removing of Pb^2+^ ions by Fe^2+^ and Fe^3+^ impregnated into PPC adsorbents. The experiments were performed in batch technique and adsorption capacity was calculated in the each step. Then, the plot of adsorption capacity via parameter changes was used to choose the optimum amount of each factor.

## Materials and methods

### Reagents

All of the chemicals used in this work were of analytical grade. The 100 ppm stock solution of Pb^2+^ was prepared by dissolving of Pb(NO_3_)_2_ in distilled water. The FeCl_3_ and FeCl_2_ solutions were used for modification of pomegranate peels. Five standard solutions of 0.0, 5.0, 10.0, 25.0, 50.0 ppm were made from a solution of 100 ppm by dilution with 1% (v/v) HNO_3_ for calibration of atomic absorption spectrometer (AAS). The working solutions with desired Pb^2+^ concentration were daily made by dilution of the stock solution.

### Preparation of composites

Pomegranate wastes were collected from Agro Industries Co in *Fars province*, *Iran*. They were dried at laboratory temperature in the absence of sunlight and sized in mesh 40–100 by standard sieves. To modify them, 10 g of the granules were mixed with 10 ml of 0.1 M Fe^3+^ and Fe^2+^ solutions for 12 h, separately. The impregnated granules were dried and then carbonized at 400 °C for 3 h into a programmable furnace in the absence of air. The residual was washed 3 times with distilled water to eject excess ions from the modified carbons. Eventually, the iron modified carbons were heated at 105 °C, named Fe^2+^-PPC and Fe^3+^-PPC and maintain for further uses.

### Calibration

The ranges of the calibration curves (0–50 ppm) for Pb^2+^ of the samples were selected for calibration of AAS. For this purpose, the absorbance of standard solutions was constructed versus concentration at a maximum wavelength of 283.3 nm. Detection and quantification limits were defined as the lowest concentrations of a component that produces a signal equal to three and ten times of the standard deviation for a series of the blank solution. The following equations were used for calculation of the detection (DL) and quantification (QL) limit:

1 $${\text{D}}.{\text{L}}. = \frac{{3.3 *\sigma {\text{A}}}}{\text{m}}$$

2 $${\text{Q}}.{\text{L}}. = \frac{{10 *\sigma {\text{A}}}}{\text{m}}$$

DL and QL are the detection and quantification limit, σ_A_ is the standard deviation of the intercept, and m is the slope of the calibration of linear equation.

### Experiments

To obtain optimal conditions, the effect of contact time, initial Pb^2+^ concentration, and adsorbent dose were determined at laboratory temperature (27 °C) based on the following experiments:

The effect of initial Pb^2+^ concentration was studied using 0.2 g of adsorbents and 50 ml of different concentrations of Pb^2+^ solution in the range of 10, 25, 50, 75 and 100 ppm in the screw-capped containers. The influence of composite dose on Pb^2+^ adsorption was examined by taking 50 ml of 50 ppm of Pb^2+^ solutions and shaking with varying amounts of adsorbents in ranging from 0.1 to 1.5 g. The similar solutions without adsorbents as control samples were examined in parallel. All of the batch experiments were performed at solution pH of 6.5 in the 150 ml closed containers and constant shaker 180 RPM using a shaker equipped with an electrical heater. At the end of all the experiments, the suspension was filtered and the concentration of Pb^2+^ in the filtered solution was measured by AAS that it was calibrated, previously. According to the obtained data, the adsorption capacity (q) of adsorbents (mg/g) was determined by the difference of initial and equilibrium concentration using the mass balance equation, as follows:

3 $$q_{t} = (C_{0} - Ce) \times \frac{V}{m}$$

Where C_0_ and C_e_ are ion concentrations (mg/l) of Pb^2+^ in solution at initial and equilibrium time, respectively, m is the mass of adsorbent (g) and V is the volume of solution (ml).

### Adsorption kinetic

For evaluation of information the kinetic adsorption mechanism, a satisfactory selection of model is required. Different adsorption models are available for designing of the adsorption rate a system. In the present study, five batch adsorption experiments were conducted by variation of contact time from 10 min up to 120 min related to kinetic studies. The obtained data were fitted to intraparticle diffusion model in order to consider the adsorption mechanism and rate limiting step in the adsorption process which expressed by Weber–Morris as follows:

4 $$q_{t} = k_{id} t^{1/2} + C_{id }$$

Where q_t_ is the adsorption capacity (mg/g), k_id_ (mg/g/min) is the intraparticle rate constant and intercept of C_id_ (mg/g) related to the thickness of the boundary layer.

### Adsorption thermodynamic

For investigation of thermodynamic parameters, the equilibrium adsorption experiments were performed by taking 50 ppm Pb^2+^ solution using 0.1 g of adsorbents at temperatures 25, 55 and 85 °C on a temperature programmable orbital shaker. The experimental data were applied to determination of thermodynamic distribution constant as follows:

5 $$K = \frac{{C_{s} }}{{C_{e} }}$$

The Gibbs free energy (ΔG), entropy (ΔS) and enthalpy (ΔH) were obtained from the Eqs. 6 and 7:

6 $$\Delta G = - RT\ln K$$

7 $${\text{lnk}} = \frac{{\Delta {\text{S}}}}{R} + \frac{{\Delta {\text{H}}}}{\text{R}} \left( {\frac{1}{T}} \right)$$

where K and R are the equilibrium and the universal gas constant (8.314 J/mol °K), C_s_ and C_e_ are ion concentrations adsorbed on the solid phase (mol/g) and in equilibrium liquid phase (mol/l), and T is the absolute temperature °K. According to Eq. 7, enthalpy function was calculated from the slope and entropy from the intercept of the line of lnK against 1/T.

## Results

### SEM

**Fig. 1:**
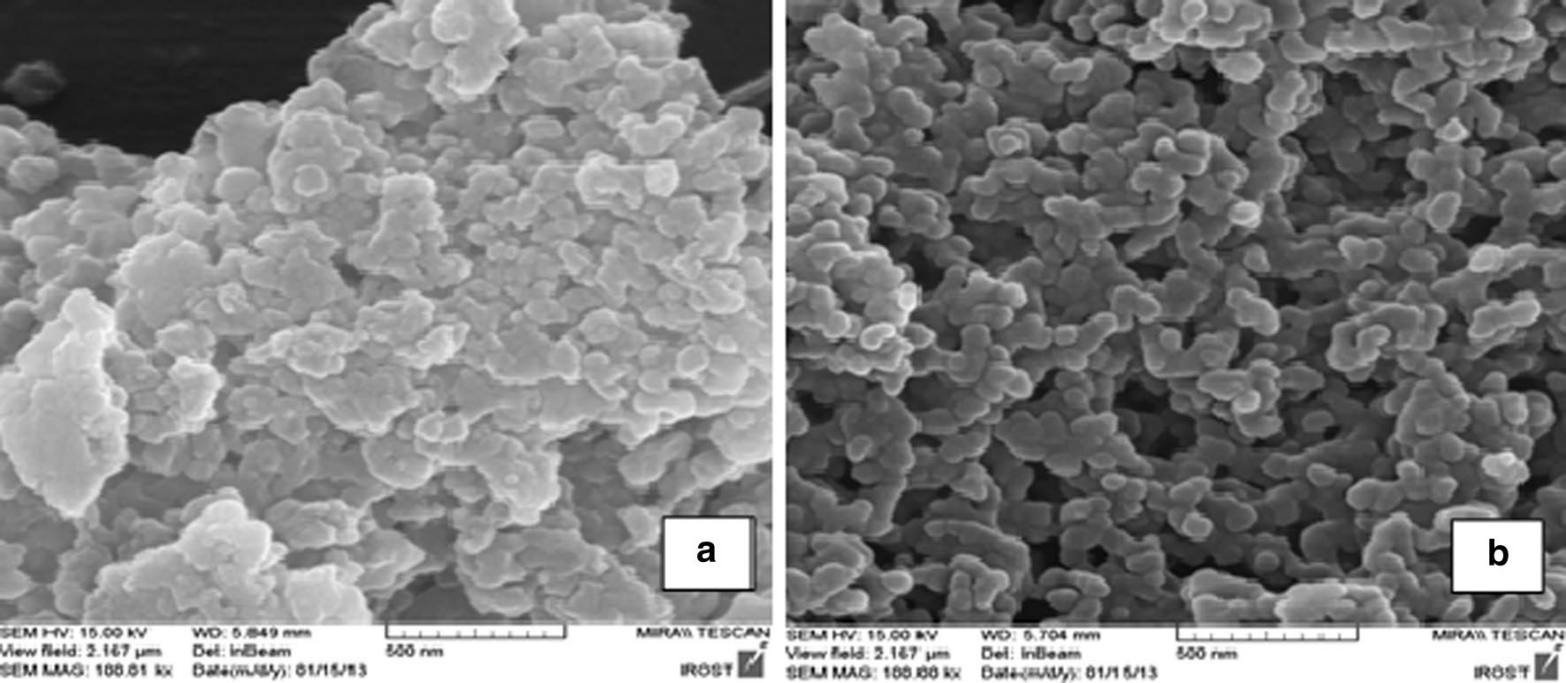
SEM images of adsorbents prepared for adsorption of Pb. SEMs were recorded in the acceleration voltage 15.00 kV and magnitude ×100.00 for focusing on surface porous. **a** SEM image of pomegranate peel carbon and **b** iron modified pomegranate peel carbon. There was a significant difference of porous between two image corresponding modifications

SEM is a powerful magnification tool that produces images of a sample by scanning the surface with a focused beam of electrons to obtain information. The electrons interact with the atoms in a sample, producing various signals that contain information about the topology of sample surface. Figure 1a, b shows the SEM photographs of the PPCs without and with impregnation of iron ions.

### FT-IR

**Fig. 2:**
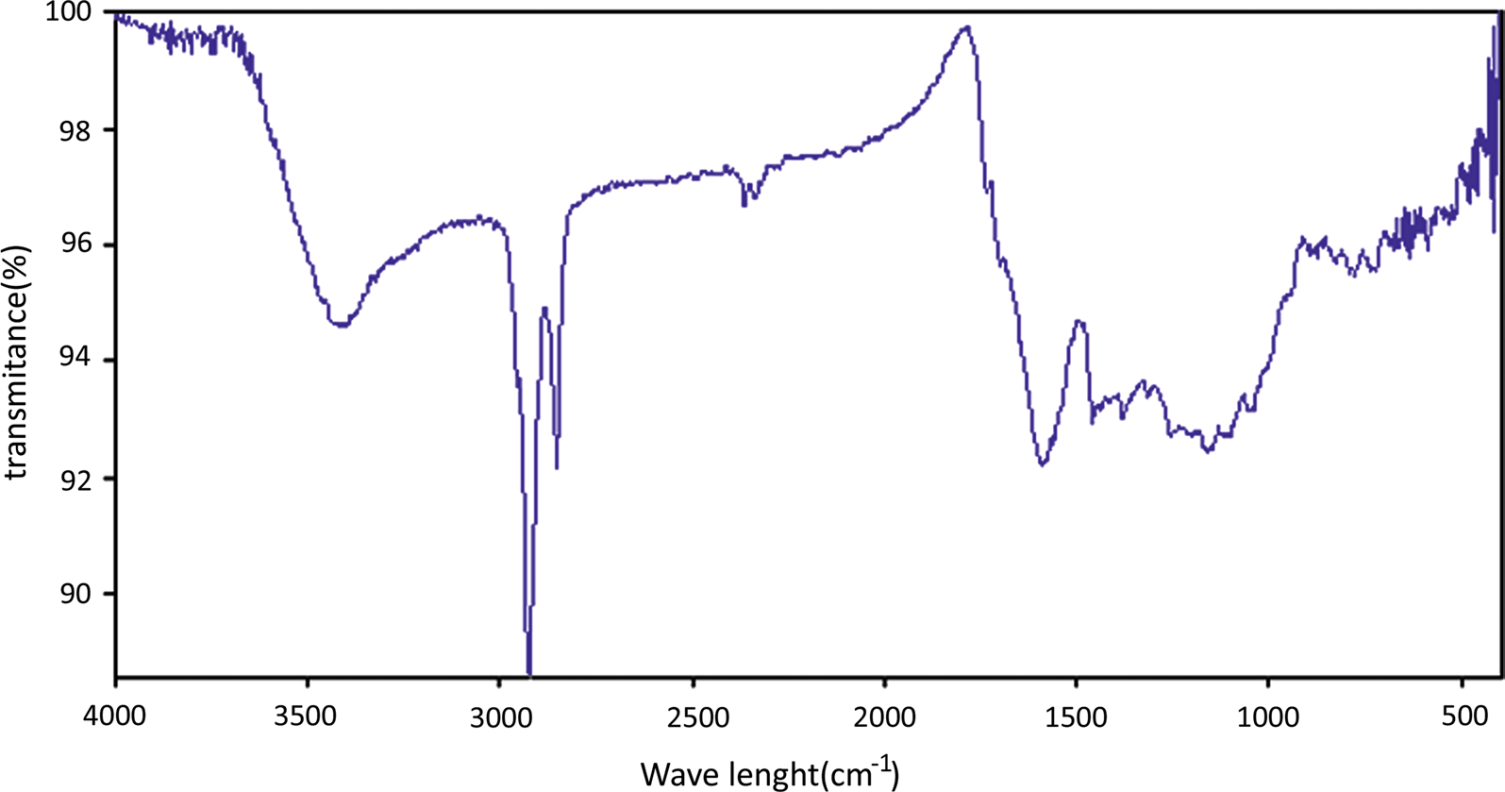
FT-IR spectrum of Fe^2+^ modified pomegranate peel carbon

FT-IR instrument is a good tool to identify the characteristic of functional groups in a compound. The main functional groups found in Fe^2+^-PPC by adsorption spectra in the infrared region, which illustrated in Fig. 2.

### Analytical sensitivity and detection limit

A calibration of absorbance versus concentration was constructed using 5 points of varying concentration. The following replicate data are presented in Table 1.

**Table 1:** The results of AA calibration for measurement of Pb^2+^

Concentration of Pb^2+^ (ppm)	No. of replicates	Mean value of signal	S D
0.0	20	0.0037	0.01108
5.0	5	0.1718	0.00227
10.0	5	0.3312	0.00368
25.0	5	0.8526	0.00225
50.0	5	1.7070	0.00574

Linearity of methods was checked within the 0–50 ppm. An analysis of the calibration data for the determination of Pb^2+^ based upon its AAS yielded the following equation:

8 $${\text{S}} = \, 0.00 3 7 { }\,\left( { \pm \, 0.0 1 1 1} \right) \, \times {\text{ C }} + \, 0.0 3 4 6 3 { }\,\left( { \pm \, 0.000 4 3 4} \right)$$

R^2^ = 0.9996 SE = 0.0132 F = 6349.17 P-value = 0.0001.

The statistical parameters show that the analytical method for determination of Pb^2+^ had suitable calibration. Sensitivity is the response of the instrument to changes in analyte concentration or a measure of the ability of method to distinguish between small differences in concentration in different samples. The sensitivity was obtained from the slope of the calibration curve. The detection limit is defined as the lowest concentration of the component, which will generate a signal/noise ratio of 3. The detection and quantification limit value were obtained from Eqs. 1 and 2. The results are represented in Table 2.

**Table 2:** Linearity, limit of detection and quantification for the AA

Parameters	Value
Linearity range	1–50 ppm
Intercept	0.0037 ± 0.0111
Slop	0.0346 ± 0.00043
Correlation coefficient (R^2^)	0.9996
Probability (P)	< 0.0001
Detection limit (D.L.)	1.050 mg/l
Quantification limit (Q.L.)	3.200 mg/l

Preliminary experiments showed that iron modified pomegranate peel carbonized at 400 °C gives the highest adsorption for Pb^2+^ ions in aqueous solution. Therefore, several batch experiments were done to find out the optimum of the effective parameters on the adsorption capacity of Fe^2+^ and Fe^3+^ impregnation to pomegranate peel carbons for adsorption of Pb^2+^ ions from equation solution.

### Effect of time

**Fig. 3:**
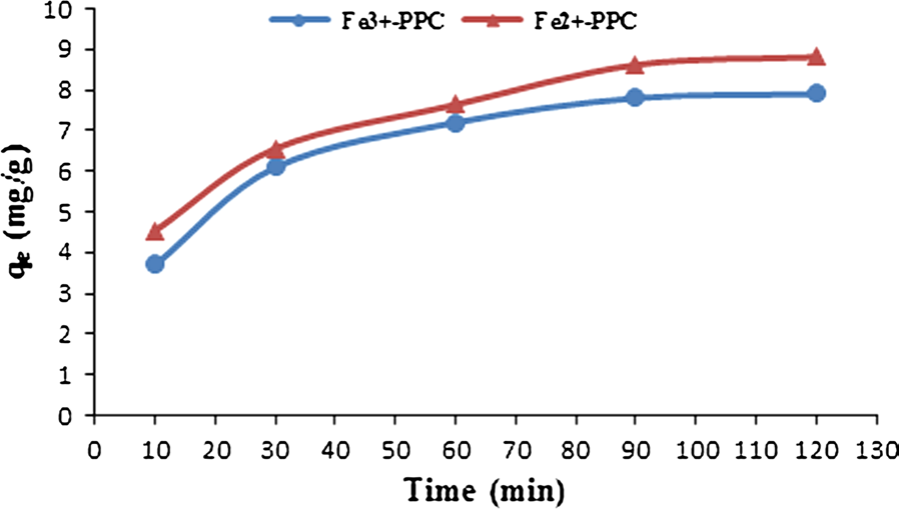
The effect of contact time on adsorption capacity. The change of adsorption capacity in removal of Pb^2+^ vs contact time for Fe^2+^-PPC is depicted in red and for Fe^3+^-PPC is depicted in blue

The results of experiments (Fig. 3) conducted to obtain the equilibrium time required for the uptake of Pb^2+^ ions by two adsorbents. For Fe^2+^-PPC and Fe^3+^-PPC, 8.8 and 8.6 mg of lead were adsorbed in the unit of adsorbent mass at the 90 min, respectively. The uptake of metal ions for Fe^2+^-PPC and Fe^3+^-PPC decreased to 8.6 and 7.9 mg/g after 120 min. In general, the total capacity adsorption of Fe^2+^-PPC and Fe^3+^-PPC was achieved constant in the 90 min. Therefore, in subsequent experiments, 90 min was selected for contact times.

### Effect of initial concentration

**Fig. 4:**
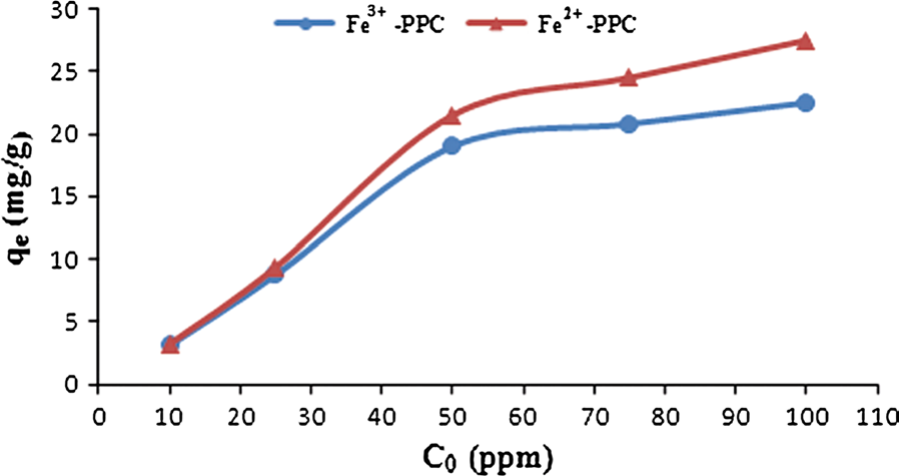
The effect of initial concentration on adsorption capacity. The change of adsorption capacity in removal of Pb^2+^ vs initial concentration of Pb^2+^ for Fe^2+^-PPC is depicted in red and for Fe^3+^-PPC is depicted in blue

Figure 4 represents the effect of initial Pb^2+^ concentrations on the adsorption capacity of Fe^2+^-PPC and Fe^3+^-PPC at pH of 6.5. The finding showed that the adsorption capacity of Pb^2+^ increased from 3.2 to 20.6 mg/g for Fe^2+^-PPC and 3.1 to 19.4 mg/g for Fe^3+^-PPC until 50 ppm, but it had a little increasing to 27.5 and 22.5 mg/g by increasing duplicate of the initial concentration of Pb^2+^ from 50 to 100 ppm.

### Effect of dose

**Fig. 5:**
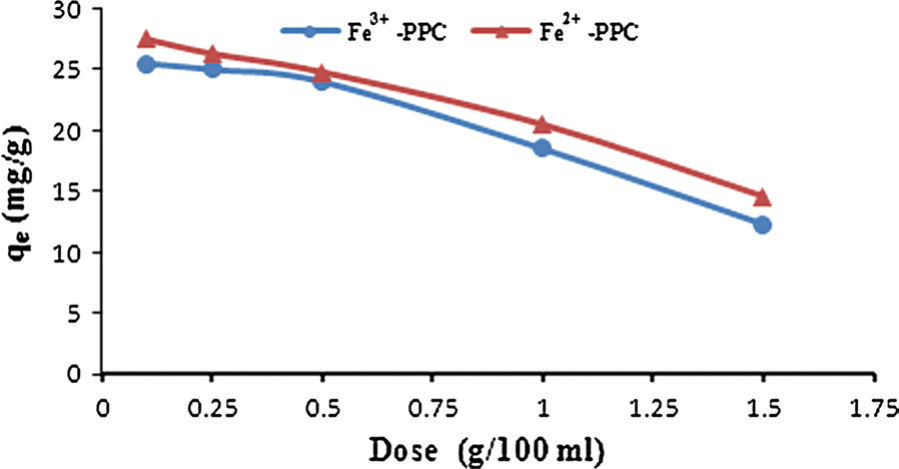
The effect of composite dose on adsorption capacity. The change of adsorption capacity in removal of the Pb^2+^ vs amount of adsorbents in solution for Fe^2+^-PPC is depicted in red and for Fe^3+^-PPC is depicted in blue

The influence of the adsorbent dose on the adsorption process at a constant initial concentration of Pb^2+^ was examined to obtain the right adsorbent mass. Figure 5 shows the plot of adsorption capacity of Fe^2+^-PPC and Fe^3+^-PPC to uptake of Pb^2+^ versus adsorbent doses. The adsorption of Pb^2+^ increased from 25.1 and 24.2 to 26.7 and 25.4 mg/g with an increase in adsorbent dose from 0.10 to 1.0 w/v% for Fe^2+^-PPC and Fe^3+^-PPC respectively. With increasing of the adsorbent dose to 1.5 w/v%, the adsorption capacity decreased from 26.7 and 25.4 to 14.6 and 13.0 mg/g further.

### Kinetic

**Fig. 6:**
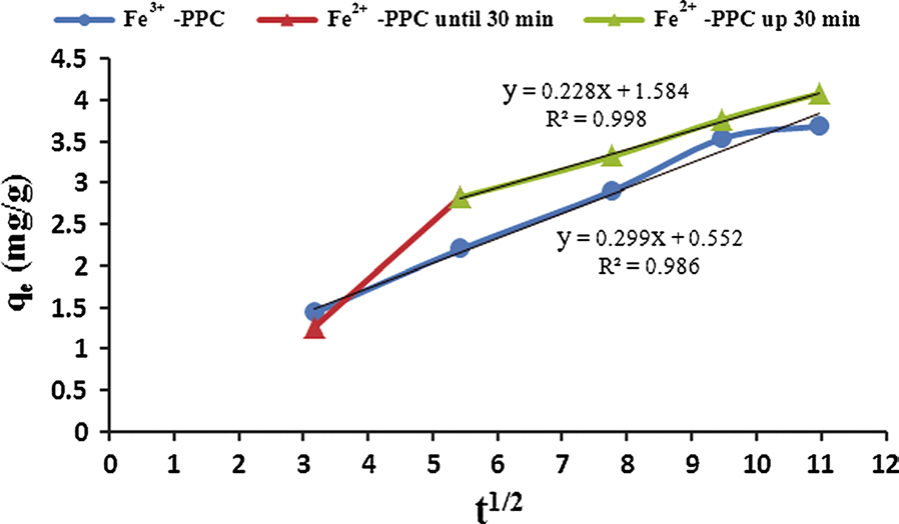
Diffusion kinetic models for Pb^2+^adsorption. The Morris Weber model kinetic were don’t in various times. This model for Fe^2+^-PPC is depicted in blue. The Morris Weber model in the range of 0–30 min for Fe^3+^-PPC is depicted in red and in the range of 30–60 min is depicted in green

In this work, the experimental data were fitted to the intraparticle diffusion equation that was given by *Weber and Morris* equation as Eq. 3. Figure 6 represents the plot of q_t_ versus t^1/2^ for Fe^2+^-PPC.

The k_id_, C_id_, and R^2^ values were obtained from the plot of q_t_ versus t^1/2^ (Fig. 6) are summarized in Table 3.

**Table 3:** The intraparticle diffusion kinetic data for adsorption of Pb^2+^ ions on iron modified pomegranate peel carbons

Adsorbent	Parameters		
	K_id_	C_id_	R^2^
	Up to 30 min		
Fe^2+^ PPC	0.228	1.584	0.998
	Total of time		
Fe^3+^ PPC	0.299	0.552	0.986

### Thermodynamic

The equilibrium constant, adsorption free energy, enthalpy, and entropy changes of reaction were estimated by Eqs. 6 and 7. The corresponding values of thermodynamic functions are summarized in Table 4.

**Table 4:** Thermodynamic parameters for Pb(II) adsorption on iron modified pomegranate peel carbons (Fe^2+^-PPC and Fe^3+^-PPC)

Temp (°C)	Fe^2+^-PPC			Fe^3+^-PPC		
	ΔG*	ΔH*	ΔS*	ΔG*	ΔH*	ΔS*
25	− 0.17			50.0		
55	− 3.87	40. 6	0.136	− 2.51	27.5	0.092
85	− 8.35			− 5.45		

* kJ/mol

## Discussion

Comparison of two images in Fig. 1 revealed that the PPC had low porosity and regular surfaces, whereas iron modified composite showed the development in their porosity and hence an increasing of the number of sites on the surface, consequently the modification with iron have been enhanced the active surface of composite.

The functional groups on the surface adsorbent are important for understanding of the adsorption process. The Fig. 2 shows a broadband about 3400 and 2900/cm which denote the presence of hydrogen-bonded OH groups on the surface of adsorbent, originally bonded to an organic polymeric structure. The IR spectra indicates a strong peaks about 1600/cm band is related to the vibration of –C=O group stretching from aldehydes, ketones, and carboxylic acids (Palin et al. 2016). The peaks of 500–900/cm regions indicate the loading of Fe^2+^ ions into functional groups of granola surface.

The pH value not only has an impact on the charge of the adsorbent surface, but also the ionization degree of particles and the existence of adsorbate species in solution (Zhan and Zhao 2003). In the case of Pb^2+^ ions, the relative hydrolysis and precipitation of Pb species at different pH, have an effect on the adsorption process with changing of the form and number of Pb ion species in solution. In aqueous solution, the general hydrolysis reaction of Pb^2+^ ions at different pH ranges can be written as follows:

9 $${\text{pH}} < 2 \quad {\text{H}}_{ 3} {\text{O}}^{ + } + {\text{ Pb}}^{ 2+ } = {\text{ no reaction}}$$

10 $${\text{pH }} = 2{-}6.5 \quad {\text{ 6H}}_{ 3} {\text{O}}^{ + } + {\text{ Pb}}^{ 2+ } = {\text{ Pb}}\left( {{\text{H}}_{ 2} {\text{O}}} \right)_{ 6}^{ 2+ } + {\text{ 6H}}^{ + }$$

11 $${\text{pH }} = 6.5{-}7 \quad {\text{6H}}_{ 2} {\text{O }} + {\text{ Pb}}^{ 2+ } = {\text{ Pb}}\left( {{\text{H}}_{ 2} {\text{O}}} \right)_{ 5} \left( {\text{OH}} \right)^{ + }_{{({\text{aq}})}} + {\text{ H}}^{ + }$$

12 $${\text{pH }} = 7{-}8 \quad {\text{2HO}}^{ - } + {\text{ Pb}}^{ 2+ } = {\text{ Pb}}\left( {\text{OH}} \right)_{{ 2({\text{aq}}.)}}$$

13 $${\text{pH}} > 8 \quad 2{\text{HO}}^{ - } + {\text{Pb}}^{2+} = {\text{Pb}} ( {\text{OH}} )_{{2({\text{s}})}} \;\; {\text{and}}\;\; {\text{then}}\;\; {\text{HO}}^{ - } + {\text{Pb}}\left( {\text{OH}} \right)_{{2({\text{s}})}} = {\text{Pb}}{{\left( {\text{OH}} \right)_{3}^{-}}}_{{({\text{aq}})}}$$

Based on the above equations, dissolved Pb species which are present in the solution, depend on pH and are in different forms such as Pb^2+^, PbOH^+^, Pb(OH)_2_, and Pb(OH)_3_^−^. The Eqs. 9 and 10 indicate that Pb^2+^ ions are the predominant species to pH of 6.5, and are responsible for maximum adsorption. Another pH conditions for the formation lead hydroxide species like PbOH^+^, Pb(OH)_2_, and ${\text{Pb}}({\text{OH}})_{3}^{-}$ occur after pH of 6.5, which causes a decrease in the adsorption yield. It is clear that precipitation of Pb^2+^ in the form of Pb(OH)_2_ at pH > 7 can be decreased the adsorption of Pb^2+^ ions, which cause a decrease in the adsorption yield. The precipitation did not occur at the pH lower than 6.5, but the adsorption yield of metal ions may be very low at acidic pH values. This situation is explained based on electrostatic repulsion forces between surface positive charges and Pb species. The Pb^2+^ ions adsorption occurred on the adsorbent surface by exchange with H^+^ at pH of 6.5.

Regards to the above, it is concluded that the pH for adsorbents must be selected higher than the pHzpc of adsorbate that it was obtained for Fe^2+^-PPC (4.3) and Fe^3+^-PPC (6.0) in our previous work (Abedi et al. 2016). In this state, most of the sorption sites on the two composites are negatively charged in the solution. In contrast, the pH for formation of Pb^2+^ must be selected lower than pH of 6.5. According to the above description, it is concluded that the adsorption process for Pb^2+^ ions will be electrically favorable at pH of 6.0–6.5. So, this pH was selected for other adsorption experiments. Under this condition, biosorption of Pb^2+^ on the Fe^2+^-PPC and Fe^3+^-PPC was expected to be favorable by electrostatic forces.

The effect of time data was studied to investigate the adsorption mechanism of Pb^2+^ ions on the iron-modified carbons. A time required for the adsorbate concentration to reach a constant value during the adsorption process was defined as adsorption equilibrium time. Figure 3 shows the rate of Pb^2+^ adsorption was fast in the initial times proportional to the enough available surface area on the adsorbent. Until time increased, more amount of Pb^2+^ adsorbed on the active sites on the adsorbent surface by attraction forces and caused a decrease in available surface areas on adsorbent (Onundi et al. 2011).

According to Fig. 4, the two states of adsorption of Pb^2+^ indicated that from 0 to 50 ppm the adsorption capacity increased proportional to the existence of unoccupied adsorption sites on both biosorbents, but from 50 to 100 ppm the adsorption sites gradually saturated and the additional concentration of Pb^2+^ remained in the solution, therefore the adsorption capacity remained nearly constant.

The Fig. 5 showed, at a constant concentration of Pb^2+^, the adsorption capacity Fe^2+^-PPC and Fe^3+^-PPC to adsorb of Pb^2+^ ions increased by increasing of the adsorbent dose. It is related to the presence of more sites on the surface of biosorbents at higher doses and the fact that certain adsorption sites are unsaturated during the batch adsorption process (Mehrasbi et al. 2009). The adsorption capacity of Fe^2+^-PPC and Fe^3+^-PPC was about marginal to 25 mg/g for two studied adsorbents when adsorbent dose increased from 0.1 to 1.0% w/v. This fall in the Pb^2+^ adsorbed amount per unit mass of biosorbent is a usual manner which has also been reported by Acharya et al. (2009). Hence, other experiments were conducted at the optimum value of 1.0% w/v of adsorbent dose.

Obtaining of the kinetic data is necessary to extend an equation for designing of the rate system and understanding the process mechanism that evaluated by matching the experimental data to kinetic models. Various kinetic models are used for evaluation of the rate adsorption process. The intraparticle diffusion model is an equation to survey the rate and mechanism of adsorption process (Srihari and Das 2008). According to them, If the line of q_t_ via t^1/2^ goes through the origin, then the limiting rate of reaction is intraparticle diffusion; as well as, if the plot forms multi-linearity, more than one stage are governed by the adsorption mechanism (Weber and Morris 1963). Figure 6 is not linear at all times, but have two regions linear. The first stage (until 30 min) indicates intraparticle diffusion or pore diffusion and the second region (up to 30 min) related to the adsorption on the interior of the adsorbent. It also indicates that the pore diffusion is not only the rate limiting mechanism and other mechanisms are involved in the adsorption of Pb^2+^ by Fe^2+^-PPC. Similar results were obtained in the study of (Jiménez-Cedillo et al. 2013). In addition, The result shows that the linearity q_t_ vs t^1/2^ plots for a total of contact time between the Pb^2+^ ions and Fe^3+^-PPC did not pass through the origin. This show that the intraparticle diffusion was only the rate-controlling step, but also the one portion linearity of the plot for Fe^3+^-PPC shows that the one stage is involved in adsorption mechanism and the boundary layer effect on the adsorption is limiting control of mechanism. According to Table 3, The value of C_id_ gives information about the boundary layer, so the value of C_id_, 1.584 for Fe^2+^-PPC and 0.552 for Fe^3+^-PPC, indicates that the effect of the boundary layer is more important in adsorption of Pb^2+^ by Fe^2+^-PPC after 30 min. These deviations from the origin may be due to the difference in the initial and the final rate of mass transfer.

Thermodynamic studies are used to predict the possibility of doing reaction in a better way and performed by changing temperature against the yield of Pb^2+^ adsorption (Huang et al. 2007). Adsorption of Pb^2+^ ions on the two composites increased by rising of the temperature from 25 to 85 °C. It is clear, increasing temperature enhances the chemical affinity and mobility of the Pb cations toward the active sites on the biosorbent surface leading to the occurrence of chemical interaction during the adsorption process. The same conclusion reported by *Garcia*-*Rosales* and *Colin*- *Cruz* in adsorption of Pb(II) and Cd(II) on the stalk sponge of *Zea mays* (García-Rosales and Colín-Cruz 2010).

The positive value of enthalpy changes (40.6 and 27.5 kJ/mol) indicates the endothermic nature of adsorption of Pb^2+^ on Fe-PPC. A possible interpretation is that the Pb^2+^ ions are hydrated in the solution. This reaction requires high energy, is endothermic, and supersedes the exothermic attraction of Pb ions toward active sites on the composite surface. The similar observation has been previously found in the removal of lead onto prepared activated carbon from coconut shell (Sekar et al. 2004). The positive value of entropy changes (136 and 92.0 J/mol) also indicates the affinity of Pb^2+^ ions toward active sites of adsorbents and suggests an increasing degree of freedom during the metal ions adsorption on an adsorbent (Arshadi, et al. 2014). The negative values of Gibbs free energy changing (ΔG), except for adsorption in the 25 °C for Fe^3+^-PPC, showed that the process is favorable and spontaneous under this experimental condition. It is also noted that the obtained ΔG values decrease by rising temperature and shows greater adsorption at the higher temperature. The negative values of Gibbs energy changing (ΔG) for Pb^2+^ adsorption conducted that the adsorption process was favorable and spontaneous. It is evident that after conversion into activated carbon and chemical modification of the low-cost adsorbents, originating of plant waste, improved the adsorption capacity of adsorbent due to the higher number of binding sites after modification and formation the new functional groups that are projected to metal ion uptake.

## Abbreviations

SEM: scanning electron microscope
FT-IR: Fourier transform infrared spectrometer
PPC: pomegranate peel carbon
Fe^2+^-PPC: iron(II) impregnated to pomegranate peel carbon
Fe^3+^-PPC: iron(III) impregnated to pomegranate peel carbon
EPA: Environmental Protection Agency
AAS: atomic absorption spectrometer
DL: detection limit
QL: quantification limit

## References

[CR1] Abedi M, Salmani MH, Mozaffari SA (2016). Adsorption of Cd ions from aqueous solutions by iron modified pomegranate peel carbons: kinetic and thermodynamic studies. Inter J Environ Sci Technol.

[CR2] Acharya J, Sahu J, Mohanty C, Meikap B (2009). Removal of lead(II) from wastewater by activated carbon developed from Tamarind wood by zinc chloride activation. Chem Eng J.

[CR3] Andrade V, Mateus M, Batoréu M, Aschner M, dos Santos AM (2015). Lead, arsenic, and manganese metal mixture exposures: focus on biomarkers of effect. Biol Trace Elem Res.

[CR4] Arshadi M, Amiri M, Mousavi S (2014). Kinetic, equilibrium and thermodynamic investigations of Ni(II), Cd(II), Cu(II) and Co(II) adsorption on barley straw ash. Water Resour Ind.

[CR5] Babel S, Kurniawan TA (2003). Low-cost adsorbents for heavy metals uptake from contaminated water: a review. J Hazard Mater.

[CR6] Cheraghi M, Sobhanardakani S, Zandipak R, Lorestani B, Merrikhpour H (2015). Removal of Pb(II) from aqueous solutions using waste tea leaves. Iranian J Toxicol.

[CR7] Ehrampoush MH, Miria M, Salmani MH, Mahvi AH (2015). Cadmium removal from aqueous solution by green synthesis iron oxide nanoparticles with tangerine peel extract. J Environ Health Sci Eng.

[CR8] García-Rosales G, Colín-Cruz A (2010). Biosorption of lead by maize (*Zea mays*) stalk sponge. J Environ Manage.

[CR9] Grant LD (2008) Lead and compounds. In: Environmental toxicants: human exposures and their health effects, 3rd edn. Wiley, Hoboken, pp 757–809

[CR10] Huang X, Gao N-Y, Zhang Q-L (2007). Thermodynamics and kinetics of cadmium adsorption onto oxidized granular activated carbon. J Environ Sci.

[CR11] Jiménez-Cedillo MJ, Olguín MT, Fall C, Colin-Cruz A (2013). As(III) and As(V) sorption on iron-modified non-pyrolyzed and pyrolyzed biomass from *Petroselinum crispum* (parsley). J Environ Manage.

[CR12] Kazemipour M, Ansari M, Tajrobehkar S, Majdzadeh M, Kermani HR (2008). Removal of lead, cadmium, zinc, and copper from industrial wastewater by carbon developed from walnut, hazelnut, almond, pistachio shell, and apricot stone. J Hazard Mater.

[CR13] Mehrasbi MR, Farahmandkia Z, Taghibeigloo B, Taromi A (2009). Adsorption of lead and cadmium from aqueous solution by using almond shells. Water Air Soil Pollut.

[CR14] Onundi YB, Mamun A, Al Khatib M, Al Saadi M, Suleyman A (2011). Heavy metals removal from synthetic wastewater by a novel nano-size composite adsorbent. Inter J Environ Sci Tech.

[CR15] Palin D, Rufato K, Linde G, Colauto N, Caetano J, Alberton O, Jesus D, Dragunski D (2016). Evaluation of Pb(II) biosorption utilizing sugarcane bagasse colonized by *Basidiomycetes*. Environ Monitor Assess.

[CR16] Pavan FA, Mazzocato AC, Jacques RA, Dias SL (2008). “Ponkan peel: a potential biosorbent for removal of Pb(II) ions from aqueous solution. Biochem Eng J.

[CR17] Renner R (2010). Exposure on tap: drinking water as an overlooked source of lead. Environ Health Pers.

[CR18] Saka C, Şahin Ö, Demir H, Kahyaoğlu M (2011). Removal of lead(II) from aqueous solutions using pre-boiled and formaldehyde-treated onion skins as a new adsorbent. Sep Sci Technol.

[CR19] Sekar M, Sakthi V, Rengaraj S (2004). Kinetics and equilibrium adsorption study of lead(II) onto activated carbon prepared from coconut shell. J Colloid Interface Sci.

[CR20] Srihari V, Das A (2008). The kinetic and thermodynamic studies of phenol-sorption onto three agro-based carbons. Desalination.

[CR21] Taha G, Arifien A, El-Nahas S (2011). Removal efficiency of potato peels as a new biosorbent material for uptake of Pb(II) Cd(II) and Zn(II) from their aqueous solutions. J Solid Waste Technol Manage.

[CR22] Weber WJ, Morris JC (1963). Kinetics of adsorption on carbon from solution. J Sanitary Eng Division.

[CR23] Wilson K, Yang H, Seo CW, Marshall WE (2006). Select metal adsorption by activated carbon made from peanut shells. Bioresour Technol.

[CR24] Zhan X-M, Zhao X (2003). Mechanism of lead adsorption from aqueous solutions using an adsorbent synthesized from natural condensed tannin. Water Res.

